# Assessment of Remineralization Treatment on Primary Enamel’s Microhardness and Mineral Composition Post Iron Drop Interaction

**DOI:** 10.1155/ijod/6637290

**Published:** 2026-01-05

**Authors:** Aneseh Sadat Tabatabaei Rad, Sara Tavassoli-Hojjati, Reyhane Sadat Hoda, Saba Aghaei

**Affiliations:** ^1^ Dental Research Center, Restorative Department, School of dentistry, Shahid Beheshti University of Medical Science, Tehran, Iran, sbmu.ac.ir; ^2^ Department of Pediatric Dentistry, Faculty of Dentistry, Tehran Medical Sciences, Islamic Azad University, Tehran, Iran, iautmu.ac.ir

**Keywords:** dental enamel, hardness, iron compounds, scanning electron microscopy, tooth remineralization

## Abstract

**Objectives:**

This study assessed the effects of remineralizing agents on microhardness and mineral content of primary enamel following iron drop exposure.

**Materials and Methods:**

In this in vitro study, 36 sound primary anterior teeth were randomly assigned to four groups (*n* = 9) of (I) casein phosphopeptide‐amorphous calcium phosphate (CPP‐ACP), (II) fluoride varnish, (III) MI varnish, and (IV) control. The microhardness of specimens was initially measured by a Vickers hardness tester. The specimens were immersed in iron drop solution in a shaker incubator at 37°C for 5 min. They were then rinsed with distilled water, and their microhardness was measured again. The teeth were subsequently split in half. The buccal halves were exposed to the remineralizing agents for 4 h, rinsed with distilled water, and immersed in artificial saliva for 24 h. They were then immersed in a demineralizing solution for 6 h, followed by a remineralizing solution for 18 h at 37°C for 10 days. The final microhardness was measured again. The buccal and lingual halves underwent energy dispersive X‐ray spectroscopy for mineral content analysis. Data were analyzed by one‐way ANOVA and Tukey test (*α* = 0.05).

**Results:**

Iron drop exposure significantly decreased, and remineralizing agents significantly increased the microhardness (both *p*  < 0.001). The three remineralizing agents had no significant difference in enhancement of microhardness (*p* = 0.493). The four groups had significant differences in Ca, F, and Fe contents after the intervention (*p*  < 0.05).

**Conclusion:**

Iron drop exposure decreased, and remineralizing agents increased the microhardness and mineral content of primary enamel under in vitro conditions.

## 1. Introduction

Reduction of primary enamel microhardness is a drawback of iron drop consumption in children [1–4]. Iron deficiency has a prevalence of 18%–38% in Iranian children under 5 years of age [5]. The adverse effects of iron deficiency anemia in children remain for years, and it can decrease the mental, physical, and behavioral functions of children [6]. Iron drops are prescribed for children to prevent this condition [1]. The majority of iron supplements contain citric acid for taste improvement; however, citric acid decreases the pH and increases enamel dissolution [5]. It can also cause dental erosion [7]. Furthermore, it enhances the enamel susceptibility to discoloration and caries by creating a rough surface [1–4, 8].

Casein phosphopeptide‐amorphous calcium phosphate (CPP‐ACP) is a dietary product that can create a super‐saturated state of calcium and phosphate in the enamel and replace the lost minerals in the tooth structure with calcium and phosphate ions, remineralizing incipient caries as such. Calcium and phosphate ions enter the enamel prisms and reform apatite crystals. Accordingly, remineralization is enhanced. CPP‐ACP has cariostatic effects and can reverse white spot lesions, incipient caries, dental erosion, root caries, and tooth hypersensitivity [9]. Moreover, professional application of fluoride can remineralize incipient enamel and white spot lesions and stop caries progression. Fluoride is used in different forms and concentrations in dentistry. Fluoride varnish is commonly used for cessation of enamel demineralization and induction of remineralization of incipient lesions in children due to its easy application and long‐term contact with demineralized enamel [10]. Long‐term exposure of enamel to fluoride varnish results in formation of calcium fluoride with a weak bond to the substrate [11]. The synergistic effect of CPP‐ACP and fluoride on enamel remineralization has been previously reported [12]. Nonetheless, the effect of remineralizing agents on enamel resistance following exposure to iron drop has not been well investigated. One previous study showed that nanohydroxyapatite‐chitosan powder increased enamel microhardness following iron drop exposure [1].

Dental caries may be prevented if remineralizing agents can strengthen the attenuated enamel structure following iron drop. Thus, this study aimed to assess the effects of fluoride varnish, CPP‐ACP, and their combination (MI varnish) on microhardness and mineral content of primary enamel following iron drop exposure.

## 2. Materials and Methods

This in vitro experimental study was conducted on 36 extracted maxillary primary anterior teeth. The study protocol was approved by the ethics committee of the university (IR.IAU.DENTAL.REC.1400.044).

### 2.1. Eligibility Criteria

Sound extracted maxillary primary anterior teeth with no caries, crack, fracture, restoration, or coronal hypoplasia were collected [13, 14]. The teeth were inspected under a stereomicroscope (Nikon LV‐TV, Japan) at x40 magnification to ensure absence of cracks. The teeth had been extracted within the past 3 months [1].

### 2.2. Sample Size

The minimum sample size was calculated to be 9 in each of the 4 groups according to a study by Mohammed et al. [15], using one‐way ANOVA power analysis of PASS 11, assuming *α* = 0.05, *β* = 0.2, and mean standard deviation and effect size = 0.62.

### 2.3. Experiment

The teeth were immersed in 0.5% chloramine T solution for 1 week, and were then stored in distilled water at 4°C until the experiment [16].

**Figure 1:**
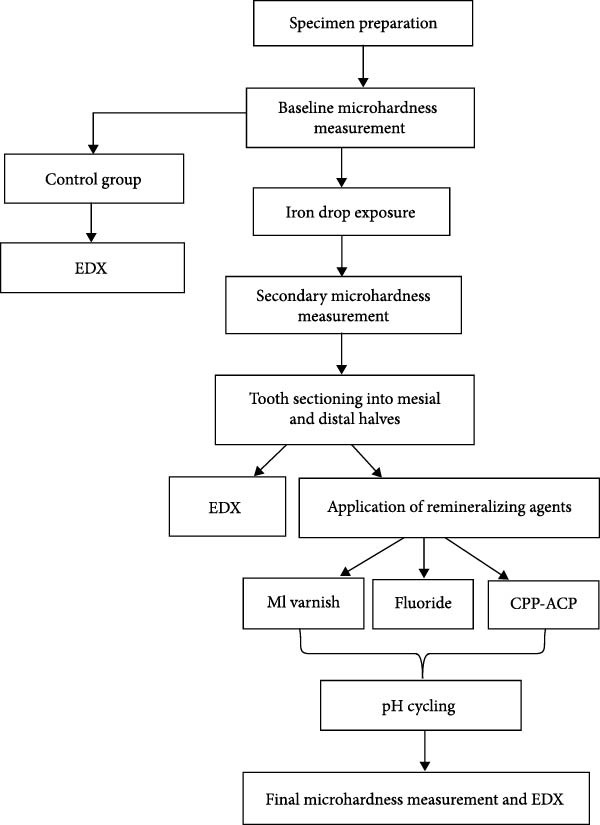
Study design flow‐diagram.

The primary enamel microhardness was measured before and after exposure to iron drop, and after the application of remineralizing agents by a Vickers hardness tester (Buehler, USA) [15, 17]. The specimens underwent the following eight steps (Figure 1):

Step I: Specimen preparation: The crowns of 36 maxillary primary anterior teeth were coated with nail varnish except for a 4 × 4 mm window on the buccal surface, and the roots were also coated with clear nail varnish [14].

**Figure 2:**
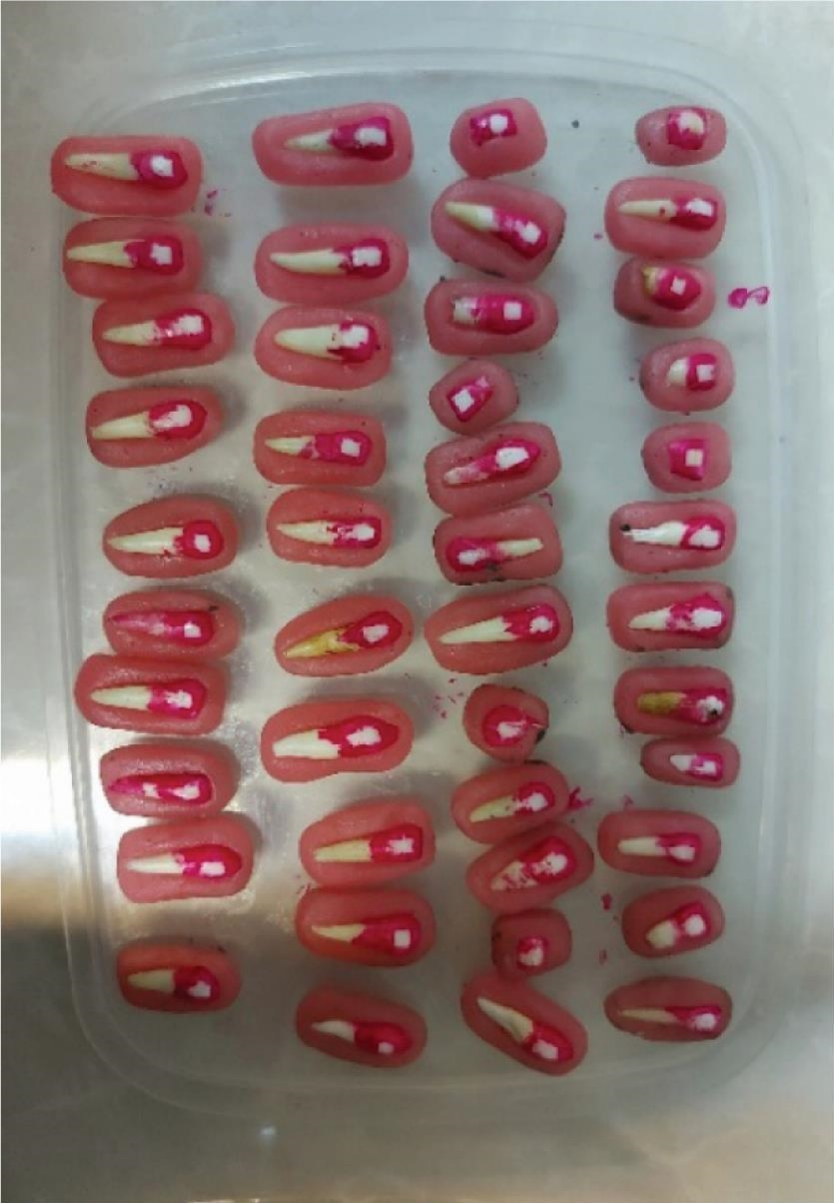
Specimens mounted for the hardness test.

Step 2: Measurement of baseline microhardness: The teeth were mounted in acrylic resin such that their lingual surface was embedded in resin, and their buccal surface remained exposed [18], as shown in Figure 2. To achieve a smooth enamel surface for indentation, minimal polishing of the buccal surface was performed by 800‐, and 2000‐grit silicon carbide abrasive papers (Lijian, China) under running water [16, 18, 19]. The buccal surface of each specimen was polished by each abrasive paper with a 90° rotation in direction compared to the direction of use of the previous abrasive paper to achieve a smooth surface with no scratches. A Vickers hardness tester (1202; Buehler, USA) was used for measurement of microhardness by applying 50 g Load for 10 s [20–22]. To measure the baseline microhardness of the specimens, a smooth part of the buccal surface was selected for load application by a diamond‐shaped indenter with a square‐shaped cross‐section. Load was automatically applied to the selected point and left a square‐shaped indentation with equal diagonals on the specimen surface. The two diagonals were measured by the device ruler under the microscope (D1 and D2) to calculate the mean value (D). The Vickers hardness number (VHN) was then calculated using the following formula:

VHN=2p  sinθ/2/D2,

where *D* is the mean diameter of the indentation (mm), *p* is the applied load (kgf), and *θ* is the angle formed between the opposing aspects of the diamond‐shaped indenter (*θ* = 136° in Vickers indenter).

To calculate the microhardness of each specimen, the VHN was measured at three points, and the mean of the three values was calculated and reported as the VHN of the specimen [23, 24]. Teeth with a VHN between 239 and 478 were selected for the study, and those with a VHN out of this range were excluded [25]. The selected teeth were immersed in distilled water at room temperature [1, 16, 26]. One control group was also considered for elemental analysis by energy‐dispersive X‐ray spectroscopy (EDX; Ametek; USA).

Step 3: Iron drop exposure: All teeth, except for the control group, were exposed to iron drop solution (Irofant ferrous sulfate, Kharazmi, Iran) at 37°C in a shaker incubator (IKA, Roentgen, Germany) for 5 min [1, 3, 18–21]. This iron drop contains 125.1 mg ferrous sulfate heptahydrate per each 1 mL (25 drops) and 25 mg sodium saccharine (as sweetener) with a pH of 1.98 [20, 21].

Step 4: Measurement of secondary microhardness: The specimens were rinsed with distilled water, and their VHN was measured again as explained for the baseline microhardness.

Step 5: Application of remineralizing agents: Each tooth was longitudinally split in half in labiolingual direction along the longitudinal tooth axis and through the midline of the created window using a diamond disk (Prodont Holliger, France). After rinsing with distilled water, one half was used for the application of remineralizing agents, and the other half was used for elemental analysis of mineral content [1, 16]. The teeth were randomly assigned to three groups (*n* = 9) using a computer‐generated table of random numbers [27]:

Group A: CPP‐ACP (Tooth Mousse; GC, USA): CPP‐ACP paste was applied on the window created in the buccal surface for 4 h [14]. It was then rinsed off with distilled water [4].
Group B: Fluoride varnish (FluoroDose; Centrix, USA): Fluoride varnish was applied on the buccal surface window for 4 h [14]. It was then removed by a cotton swab dipped in acetone and rinsed off with distilled water [14].
Group C: Combination of CPP‐ACP and fluoride varnish (MI varnish; GC, USA): MI varnish was applied on the window for 4 h. It was then removed with a cotton swab dipped in acetone and rinsed off with distilled water [14]

After the application of remineralizing agents, the specimens were immersed in artificial saliva composed of 500 mL of distilled water, 20 g xylitol, 1.2 g potassium chloride, 0.843 g sodium chloride, 0.051 g magnesium chloride, 10 g sodium carboxymethyl cellulose, and 20 mL of stock solution including 1% tricalcium phosphate and 0.05 mL sodium hydroxide for pH adjustment [14].

Step 6: pH cycling: All specimens were subjected to a remineralization‐demineralization cycle. The demineralizing solution included 2.2 mmol Ca(NO_3_)2, 2.2 mmol KH_2_PO_4_, 0.1 ppm NaF, and 50 mmol acetic acid. The remineralizing solution included 1.5 mmol CaCl_2_, 0.9 mmol KH_2_PO_4_, 130 mmol KCl, and 20 mmol Hepes with a pH of 7. The teeth were immersed in the demineralization solution for 6 h, and in the remineralization solution for 18 h at 37°C for 10 consecutive days [14]. The solutions were replaced daily [4].

Step 7: Measurement of final microhardness: The final microhardness of the specimens was measured again as explained for baseline and secondary microhardness in a blind manner. Finally, the percentage of change in VHN of each specimen was calculated.

**Figure 3:**
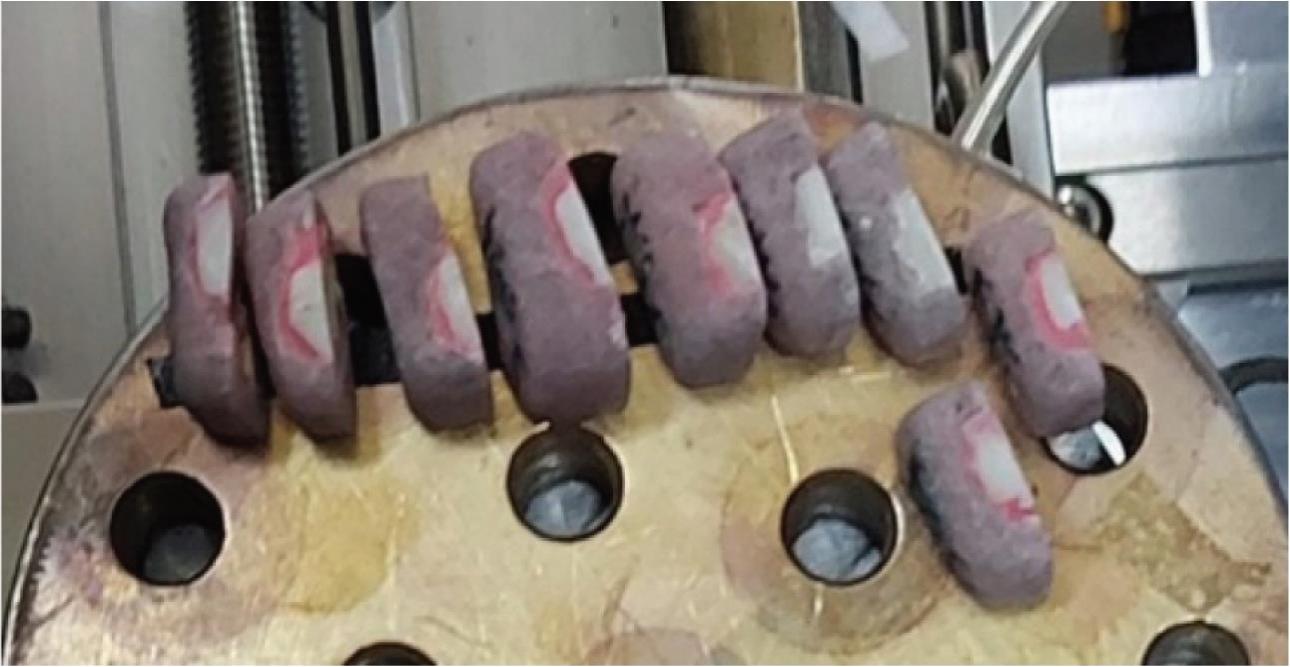
Specimens mounted for EDX analysis.

Step 8: Mineral content analysis: Tooth haves were rinsed with deionized water, mounted in copper, and underwent EDX (Ametek, USA) (Figure 3). The elemental content of calcium (Ca), phosphorus (P), fluoride (F), and iron (Fe) in atomic percentage was determined according to the graph provided by the respective software. The Ca, P, F, and Fe contents were reported as the Ca/P ratio for each group [14].

### 2.4. Statistical Analysis

The amount of different elements and changes in microhardness in the four groups were analyzed by one‐way ANOVA. Considering the normal data distribution as confirmed by the Kolmogorov–Smirnov test, and homogeneity of variances, Tukey’s test was used for pairwise comparisons. *p*  < 0.05 was considered statistically significant.

## 3. Results

**Figure 4:**
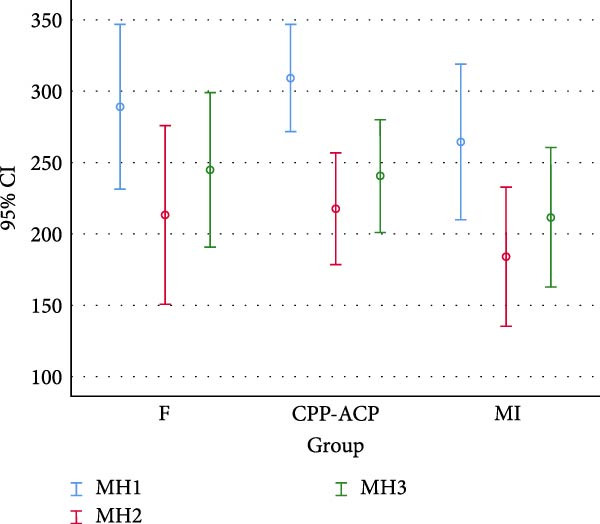
Microhardness of the groups at three steps.

Table 1 and Figure 4 present the baseline, secondary, and final enamel microhardness and the results of EDX analysis in the four groups. Considering the normal distribution of data as confirmed by the Kolmogorov–Smirnov test (*p*  > 0.05), one‐way ANOVA revealed a significant difference in baseline, secondary, and final microhardness in all three experimental groups (*p*  < 0.001). Within‐group comparison of microhardness revealed significant differences in all three steps (*p*  < 0.001), such that the secondary microhardness significantly decreased following iron drop exposure (*p*  < 0.05) and the final microhardness significantly increased after the application of remineralizing agents (*p*  < 0.001).

**Table 1:** Baseline, secondary, and final enamel microhardness (VHN) and mineral content in the four groups.

Tests groups	Mean ± SD Microhardness (kgf/mm^2^)			*p* value multiple comparisons Tukey HSD	Mean ± SD mineral content (wt%)								*p* value paired samples test
	Mh1^a^	Mh2^b^	Mh3^c^		Ca	CaH	P	PH	F	FH	Fe	FeH	
Control	305.32 ± 77.65	—	—	—	70.69 ± 1.26	—	28.59 ± 1.11	—	0.17 ± 0.20	—	0.53 ± 0.25	—	—
FV	286.42 ± 23.41	315.23 ± 28.39	136.58 ± 15.34	Mh1–Mh2:<0.0001 ^∗^ Mh1–Mh3:<0.0001 ^∗^ Mh2–Mh3:<0.0001 ^∗^	69.15 ± 2.56	66.76 ± 2.839	28.12 ± 3.90	27.80 ± 2.79	0.64 ± 0.43	0.40 ± 0.44	2.07 ± 1.49	5.02 ± 2.09	Ca‐CaH^#^:0.009 P‐PH^#^:0.819 F‐FH^#^:0.294 Fe‐FeH^#^:0.021
CPP‐ACP	288.95 ± 80.92	213.17 ± 87.52	244.76 ± 75.73	Mh1–Mh2:<0.0001 ^∗^ Mh1–Mh3:<0.0001 ^∗^ Mh2–Mh3:<0.0001 ^∗^	67.27 ± 1.44	68.12 ± 1.83	30.03 ± 1.82	26.09 ± 3.96	0.25 ± 0.36	0.41 ± 0.66	2.35 ± 0.99	5.36 ± 3.58	Ca‐CaH:0.020 P‐PH:0.266 F‐FH:0.255 Fe‐FeH:0.023
MI varnish	267.91 ± 62.64	298.12 ± 58.60	128.62 ± 8.09	Mh1–Mh2:<0.0001 ^∗^ Mh1–Mh3:<0.0001 ^∗^ Mh2–Mh3:<0.0001 ^∗^	68.88 ± 1.32	66.50 ± 2.50	28.76 ± 1.36	29.51 ± 0.99	0.09 ± 0.20	0.44 ± 0.53	2.37 ± 1.06	4.02 ± 2.14	Ca‐CaH, 0.002 ^∗^ P‐PH: 0.032 ^∗^ F‐FH:0.098 Fe‐FeH, <0.0001 ^∗^
*p* value	0.41	0.41	0.41	—	0.004	—	0.387	—	0.006	—	0.003	—	—

*Note*: Mineral content was measured after the application of the remineralizing agent in the control group and after the application of remineralizing agent +iron drop in the study groups.

^a^Mh1: first microhardness.

^b^Mh2: microhardness following application of iron drop.

^c^Mh3: microhardness following exposure to remineralizing agent.

^∗^Significant.

^#^CaH/FeH/PH/FH: Ca, Fe, P, and F contents in dental halves of the study group.

Between‐group comparisons showed no significant difference in baseline microhardness of the study groups (*p* = 0.540). The final microhardness of the groups was not significantly different either (*p* = 0.493), indicating no significant difference in efficacy of remineralizing agents in enhancement of microhardness.

**Figure 5:**
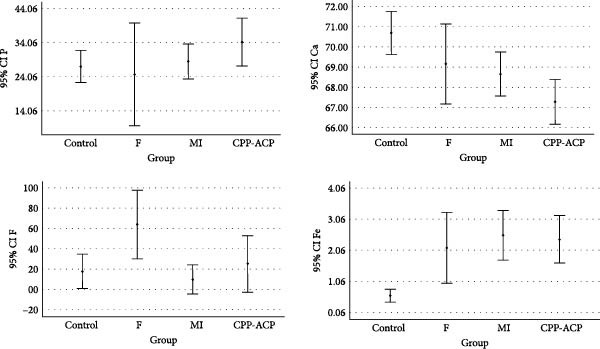
Fe, F, P, and Ca contents in the four groups according to EDX results.

Figure 5 shows the Fe, F, P, and Ca contents in the four groups according to the EDX results. One‐way ANOVA showed a significant difference among the groups in Ca (*p*  = 0.004), F (*p*  = 0.006), and Fe (*p* = 0.003) contents, but not in P content (*p*  > 0.05) after the application of remineralizing agents. Pairwise comparisons by the Tukey’s test revealed a significant difference in Ca content only between the control and CPP‐ACP groups (*p* = 0.002). The F content in fluoride varnish and MI varnish groups was significantly higher than that in the control and CPP‐ACP groups (*p* = 0.006). The Fe content was significantly different between the control and MI varnish (*p* = 0.004), control and CPP‐ACP (*p* = 0.007), control and fluoride varnish (*p* = 0.026), and fluoride varnish and MI varnish (*p* = 0.006) groups; however, the difference in this regard was not significant among CPP‐ACP, fluoride varnish, and MI varnish groups (*p* = 0.851).

Comparison of elements between the remineralized half and the other half (only exposed to iron drop) by paired sample test revealed that the Ca content of the remineralized half was higher than that of the other half (exposure to iron drop only), and the Fe content of the remineralized half was lower than that of the other half. In other words, in fluoride group, the difference in Ca (*p* = 0.009) and Fe (*p* = 0.021) contents was significant between the halves. Also, in MI varnish group, the difference in Ca (*p* = 0.047) was significant between the halves in spite of Fe (*p* = 0.066). In the CPP‐ACP group, the difference in Ca (*p* = 0.02) and Fe (*p* = 0.023) contents was also significant between the halves, such that the Ca content was higher in halves subjected to iron drop and remineralizing agents, while the Fe content was higher in halves exposed to iron drop only. Finally, the Ca content was almost higher and the Fe content was lower in remineralized halves of the experimental groups compared with their other half exposed to iron drop alone.

## 4. Discussion

Dental erosion has ~3 times higher prevalence in primary enamel than permanent enamel due to incomplete mineralization, and iron drop is a common cause of primary enamel erosion [18]. Thus, this study assessed the effects of fluoride varnish, CPP‐ACP, and their combination (MI varnish) on microhardness and mineral content of primary enamel following iron drop exposure. The present results showed that iron drop exposure significantly decreased primary enamel microhardness, which was in line with the findings of previous studies [1, 3, 28]. Application of remineralizing agents significantly increased primary enamel microhardness and compensated for the reduction in microhardness due to iron drop exposure to some extent, which was in line with the results of Tabari et al. [1], who showed that application of nanohydroxyapatite remineralizing agent after iron drop exposure increased enamel microhardness. Another study also showed an increase in primary enamel microhardness by the application of remineralizing agents [18]. However, Maurya et al. [29] indicated that application of MI varnish following immersion of primary teeth in several commonly used children medications could not significantly prevent the reduction in enamel microhardness, which may be due to the short and probably insufficient exposure time of the teeth to MI varnish, which was 1 min in their study. Iron drops contain citrate acidic compounds that cause porosities in the enamel surface, compromise the structure of hydroxyapatite, and cause erosion, resulting in a reduction in microhardness [18, 30]. Subsequently, application of remineralizing agents containing fluoride and calcium and phosphorus ions reinforces the structure of hydroxyapatite by the formation of fluorapatite, strengthens the superficial enamel, and enhances its microhardness [28]. The role of fluoride and CPP‐ACP in enhancement of remineralization and reduction of demineralization has been previously confirmed [31–33]. Microscopic porosities in the enamel surface enhance absorption of fluoride and other ions [34].

The second objective of the present study was to analyze the mineral content of primary enamel and its alterations following exposure to different materials by EDX, which is a highly accurate method for this purpose [12]. Following exposure to iron drops, which are acidic and have a pH of 2–5, the pH of the oral environment drops, and the teeth become susceptible to erosion. Resultantly, calcium and phosphorus ions are released from the tooth structure to neutralize the oral environment [35, 36]. Subsequently, Fe ions present in iron drop fill the empty spaces of the released Ca and P ions in the superficial enamel structure, and react with the enamel surface phosphate ions, forming ferric phosphate, which deposits on the tooth surface [34, 37]. Evidence shows that calcium and phosphate compounds are more stable than ferric sulfate in neutral environments; thus, in the neutralized oral environment, new calcium ions present in remineralizing agents replace the released ferric ions and form stable calcium and phosphate compounds [34, 37]. The present EDX results, according to Figure 5, revealed higher Ca content in remineralized halves compared with the other halves (iron drop exposure only). In other words, application of remineralizing agents, including fluoride varnish, MI varnish, and CPP‐ACP (close to significant), increased Ca uptake by the primary enamel, which was in agreement with the EDX results in a study by Salman et al. [14]. In other words, by release of Fe and its replacement with Ca present in the remineralizing agent, the Ca content increases and a more stable structure is formed. The MI varnish group showed the highest increase in Ca ion content, which was in agreement with the findings of Salman et al. [14]. Also, the Fe content significantly decreased in all groups (close to significant in MI varnish group) after the application of remineralizing agents, confirming the abovementioned statements. In other words, application of remineralizing agents decreased iron uptake by the primary enamel.

The F content in F and MI varnish groups was significantly higher than that in the control and CPP‐ACP groups. Other studies also reported a significant increase in F content following the application of fluoridated varnishes such as fluoride varnish and CPP‐ACPF in the enamel surface, which was in line with the present results [36, 38, 39].

The present study also sought to assess whether application of remineralizing agents can compensate for the adverse effect of iron drop exposure on enamel microhardness. As mentioned earlier, the adverse effect of iron drop on reduction of enamel microhardness and increasing its susceptibility to erosion and caries has been confirmed [40]. However, Ca, P, and F ions of remineralizing agents enter the apatite structure and reform the crystals, and fill the empty spaces in a stable manner [37, 41, 42]. Thus, application of remineralizing agents can increase enamel microhardness and mineral content and can compensate, at least partly, for the reduction in microhardness caused by iron drop exposure with a pH of ~1.9 [20].

This study had an in vitro design and was conducted on sound extracted primary anterior teeth. Thus, generalization of results to the clinical setting and other tooth types must be done with caution. Future studies are required to take into account the effect of confounding factors such as viscosity of iron drop, presence of white spot lesions on primary teeth, and oral flora of children.

## 5. Conclusion

Within the limitations of this in vitro study, the results showed that iron drop exposure decreased primary enamel microhardness and Ca content and increased Fe content, while application of fluoride varnish, CPP‐ACP, and MI varnish remineralizing agents equally increased the microhardness, increased the Ca content, and decreased the Fe content of enamel.

## Conflicts of Interest

The authors declare no conflicts of interest.

## Funding

This research did not receive any specific grant from funding agencies in the public, commercial, or not‐for‐profit sectors.

## Data Availability

The data used to support the findings of this study were supplied by corresponding author under license, and data will be available on request. Requests for access to these data should be made to corresponding author.
